# Determination of MIC Distribution of Arbekacin, Cefminox, Fosfomycin, Biapenem and Other Antibiotics against Gram-Negative Clinical Isolates in South India: A Prospective Study

**DOI:** 10.1371/journal.pone.0103253

**Published:** 2014-07-28

**Authors:** Sangeetha Rajenderan, Veeraraghavan Balaji, Shalini Anandan, Rani Diana Sahni, Giannoula S. Tansarli, Matthew E. Falagas

**Affiliations:** 1 Christian Medical College, Vellore, Tamil Nadu, South India; 2 Alfa Institute of Biomedical Sciences, Athens, Greece; 3 Department of Internal Medicine - Infectious Diseases, Iaso General Hospital, Iaso Group, Athens, Greece; 4 Department of Medicine, Tufts University School of Medicine, Boston, Massachusetts, United States of America; University Medical Center Utrecht, Netherlands

## Abstract

**Objectives:**

To determine the *in vitro* activity of antibiotics, including arbekacin, cefminox, fosfomycin and biapenem which are all still unavailable in India, against Gram-negative clinical isolates.

**Methods:**

We prospectively collected and tested all consecutive isolates of *Escherichia coli, Klebsiella* spp., *Pseudomonas aeruginosa* and *Acinetobacter* spp. from blood, urine and sputum samples between March and November 2012. The minimum inhibition concentration (MIC) of 16 antibiotics was determined by the broth micro-dilution method.

**Results:**

Overall 925 isolates were included; 211 *E*. *coli*, 207 *Klebsiella* spp., 153 *P*. *aeruginosa*, and 354 *Acinetobacter* spp. The MIC_50_ and MIC_90_ were high for cefminox, biapenem and arbekacin for all pathogens but interpretative criteria were not available. The MIC_50_ was categorized as susceptible for a couple of antibiotics, including piperacillin/tazobactam, carbapenems and amikacin, for *E*. *coli*, *Klebsiella* spp. and *P*. *aeruginosa*. However, for *Acinetobacter* spp., the MIC_50_ was categorized as susceptible only for colistin. On the other hand, fosfomycin was the only antibiotic that inhibited 90% of *E*. *coli* and *Klebsiella* spp. isolates, while 90% of *P*. *aeruginosa* isolates were inhibited only by colistin. Finally, 90% of *Acinetobacter* spp. isolates were not inhibited by any antibiotic tested.

**Conclusion:**

Fosfomycin and colistin might be promising antibiotics for the treatment of infections due to *E*. *coli* or *Klebsiella* spp. and *P*. *aeruginosa*, respectively, in India; however, clinical trials should first corroborate the *in vitro* findings. The activity of tigecycline should be evaluated, as this is commonly used as last-resort option for the treatment of multidrug-resistant *Acinetobacter* infections.

## Introduction

Antimicrobial resistance has risen alarmingly worldwide during the last decade. The widespread of Gram-negative organisms producing extended-spectrum beta-lactamases (ESBLs) conferring resistance to penicillins, cephalosporins, and fluoroquinolones, or carbapenemases conferring resistance even to carbapenems limits significantly the treatment armamentarium against infections. India is one of the countries facing the greatest burden of antimicrobial resistance around the world. The high availability of antibiotics over the counter in the country is major contributor in the high antimicrobial resistance observed. New-Delhi Metallo-β-lactamase (NDM)-producing Enterobacteriaceae, which were first detected here, [1] are now endemic in India. [2] Carbapenemase-producing Enterobacteriaceae cause difficult-to-treat infections usually characterized by high mortality. [3], [4] Furthermore, high prevalence of infections caused by carbapenemase-producing *Acinetobacter baumannii* [5], [6], [7] and *Pseudomonas aeruginosa*, [6], [8] as well as of infections caused by ESBL-producing Enterobacteriaceae has been observed in India [9], [10], [11].

The introduction into clinical practice of antibiotics that are still unavailable in India could be a solution to the problem of the antimicrobial resistance. Arbekacin, the cephamycin cefminox, and the group 2 carbapenem biapenem are effective antibiotics, mainly used in Japan and South Korea. Arbekacin is primarily used for the treatment of infections caused by methicillin-resistant *Staphylococcus aureus* [12] and few *in vitro* data suggest that this antibiotic might be also considered as an adjunct treatment for infections due to multidrug-resistant (MDR) Gram-negative pathogens., [13], [14] Cefminox is active against anaerobic bacteria [15] as well as ESBL-producing Enterobacteriaceae. [16] As regards biapenem, this is active against Gram-negative and Gram-positive anaerobic bacteria, [17], [18] but also against aerobic bacteria, both alone [19] and in combination with other agents against MDR pathogens. [20] Finally, fosfomycin, which is also not marketed in India, is an “old” antibiotic, discovered in the late 60’s which has been re-evaluated the last years and re-introduced successfully into clinical practice in many countries of the world. [21], [22], [23] Fosfomycin has broad antimicrobial spectrum against MDR pathogens, both Gram-negative [24] and Gram-positive ones [25].

In this context, we aimed to determine the minimum inhibition concentration (MIC)_50_ (the antibiotic concentration required to inhibit the growth of 50% of the pathogens) and MIC_90_ (the antibiotic concentration required to inhibit the growth of 90% of the pathogens) of antibiotics, including arbekacin, cefminox, biapenem and fosfomycin for common Gram-negative clinical isolates collected from patients with hospital- or community-acquired infections in a tertiary care hospital, in South India.

## Methods

### Study design and setting

This prospective study was performed at the Christian Medical College, Vellore, South India, at the Department of Clinical Microbiology, between March and November 2012. All consecutive isolates of *E*. *coli*, *Klebsiella* spp., *P*. *aeruginosa*, and *Acinetobacter* spp. isolated from the urine, blood, and sputum were included in the study. Only one isolate per patient was included in the study. Data on the demographic details of patients whose isolates were studied was not included.

### Ethics considerations

The study was approved by the Institutional review board and ethics committee of Christian Medical College, Vellore, South India. A written or oral informed consent was not obtained by the patients whose isolates were included in this study due to the non-interventional study design and this consent procedure was approved by the ethics committee of our institution.

### Microbiological methods

Isolation and identification of the isolates from the specimens was performed using a semi-quantitative culture method and biochemically characterized using the mannitol motility medium, triple sugar iron agar medium, peptone water and Simmons citrate medium. [26], [27] Identification up to the genus level for *Klebsiella* and *Acinetobacter* isolates was performed. An oxidase test was also performed for *Acinetobacter* spp. and *P*. *aeruginosa* and an indole test was performed in order to differentiate between *Klebsiella* spp. and *E*. *coli*. Standard American type culture collection (ATCC) control strains within acceptable limits were used as quality control strains for the drugs tested. *E*. *coli* ATCC 25922, *Staphylococcus aureus* ATCC 29213, *P*. *aeruginosa* ATCC 27853 and *Enterococcus faecalis* ATCC29212 were used for susceptibility testing to ampicillin/sulbactam, piperacillin/tazobactam, cefminox, cefmetazole, ceftazidime, ceftriaxone, aztreonam, fosfomycin, imipenem, meropenem, doripenem, biapenem, amikacin, arbekacin, gentamicin and colistin.

The MIC was determined by the broth micro-dilution method (Meiji Co., Japan) using cation-adjusted Muller-Hinton broth. The inoculum was prepared by the growth method with which the test bacteria were grown on non-selective culture media and incubated overnight. On the following day, 4–5 colonies were taken from that plate and suspended into 2 ml of nutrient broth and incubated for 2 hours. The bacterial inoculum was adjusted to 1 McFarland Standard by sterilized physiological saline. Then, 25 µL of the inoculum was added into 12 mL of cation-adjusted Mueller-Hinton broth (CAMHB) and 50 µL of the mixture was inoculated into each plate. The final inoculum size was approximately 2.5×10^4^ CFU of bacteria in each plate. Finally, the inoculated plates were incubated at 35±2°C in ambient air for 20–24 hours for *Acinetobacter* spp. or 16–20 hours for the other bacteria.

### Definitions and data analysis

The MIC range, MIC_50_, and MIC_90_ were determined for cefminox, arbekacin, fosfomycin biapenem, ampicillin/sulbactam, piperacillin/tazobactam, cefmetazole, ceftazidime, ceftriaxone, aztreonam, imipenem, meropenem, doripenem, amikacin, gentamicin and colistin for the 4 pathogens. The interpretation of the MIC_50_ and MIC_90_ was performed using the Clinical and Laboratory Standards Institute (CLSI) 2012 guidelines. [28] The MICs for fosfomycin were reported as susceptible (≤64 µg/ml), intermediate (128 µg/ml), or resistant (≥256 µg/ml). Interpretative criteria of the MIC are not available by CLSI for cefminox, biapenem and arbekacin for any of the four pathogens tested and for fosfomycin for *P*. *aeruginosa* and *Acinetobacter* spp.

## Results

A total of 925 isolates were collected and tested during the study period; 211 isolates of *E*. *coli*, 207 of *Klebsiella* spp., 153 of *P*. *aeruginosa*, and 354 of *Acinetobacter* spp. With regard to the source of isolation, 363 originated from the sputum, 362 isolates from the blood, and 200 isolates from the urine. 74% and 75% of the isolates identified in the blood and urine, respectively, were Enterobacteriaceae, while 87.9% and 12.1% of the isolates identified in the sputum were *Acinetobacter* and *P*. *aeruginosa*, respectively. The pathogens by source of isolation are presented in Table 1. The isolates originated from medical, surgical, and critical care departments of the hospital.

**Table 1:** Pathogens by source of isolation.

Pathogen	Sputum	Blood	Urine	Total
*Escherichia coli*	-	131	80	211
*Klebsiella* spp.	-	137	70	207
*Pseudomonas aeruginosa*	44	59	50	153
*Acinetobacter* spp.	319	35	-	354
**Total**	363	362	200	925

### E. coli

The MIC range, MIC_50_, and MIC_90_ of all antibiotics tested are presented in Table 2. The MIC_50_ was low for piperacillin/tazobactam, cefmetazole, fosfomycin, imipenem, meropenem, doripenem, and amikacin (8/4, 1, 0.5, 0.12, ≤0.06, ≤0.03, and 2 µg/ml, respectively). These values are categorized as susceptible by CLSI. The only antibiotic with low MIC_90_, categorized as susceptible, was fosfomycin (0.5 µg/ml). Both MIC_50_ and MIC_90_ were low for colistin, 0.25 and 0.5 µg/ml respectively, while low MIC_50_ was found for cefminox, biapenem, and arbekacin (1, ≤0.06, and 2 µg/ml, respectively). However, interpretative criteria were not available for any of these antibiotics.

**Table 2:** Determination and interpretation of the MIC values of the antibiotics tested for *Escherichia coli* (n = 211).

Antibiotic	MIC range	MIC_50_ (µg/ml)	Interpretation*	MIC_90_ (µg/ml)	Interpretation*
Amp/sulb	0.5/0.25–>256/128	16/8	I	>256/128	R
Pip/taz	≤0.06/4–>128/4	8/4	S	>128/4	R
Cefminox	≤0.06–>128	1	No criteria	128	No criteria
Cefmetazole	0.12–>128	1	S	>128	R
Ceftazidime	≤0.03–>64	16	R	>64	R
Ceftriaxone	≤0.06–>128	>128	R	>128	R
Aztreonam	≤0.06–>128	32	R	>128	R
Fosfomycin	≤0.25–>256	0.5	S	4	S
Imipenem	≤0.03–>64	0.12	S	64	R
Meropenem	≤0.06–>128	≤0.06	S	64	R
Doripenem	≤0.03–>64	≤0.03	S	64	R
Biapenem	≤0.06–>128	≤0.06	No criteria	16	No criteria
Amikacin	≤0.06–>128	2	S	>128	R
Arbekacin	≤0.06–>128	2	No criteria	>128	No criteria
Gentamicin	≤0.06–>128	16	R	>128	R
Colistin	0.06–>32	0.25	No criteria	0.5	No criteria

*The interpretation of the MIC_50_ and MIC_90_ of all antibiotics tested for all pathogens was performed using the Clinical and Laboratory Standards Institute (CLSI) 2012 guidelines.

**Abbreviations:** S: susceptible, R: resistant, I: intermediate, amp/sulb: ampicillin/sulbactam, pip/taz: piperacillin/tazobactam, MIC: minimum inhibition concentration.

### *Klebsiella* spp

The MIC range, MIC_50,_ and MIC_90_ of all antibiotics tested are presented in Table 3. The MIC_50_ for piperacillin/tazobactam, cefmetazole, fosfomycin, imipenem, meropenem, doripenem, and amikacin was low, categorized as susceptible (4/4, 2, 8, 0.25, ≤0.06, 0.06, and 2 µg/ml, respectively). Fosfomycin was the only antibiotic with low MIC_90_ (32 µg/ml), within the susceptible range.

**Table 3:** Determination and interpretation of the MIC values of the antibiotics tested for *Klebsiella* spp. (n = 207).

Antibiotic	MIC range	MIC_50_ (µg/ml)	Interpretation*	MIC_90_ (µg/ml)	Interpretation*
Amp/sulb	0.5/0.25–>256/128	16/8	I	>256/128	R
Pip/taz	≤0.06–>128	4/4	S	>128/4	R
Cefminox	≤0.06–>128	1	No criteria	>128	No criteria
Cefm\etazole	≤0.06–>128	2	S	>128	R
Ceftazidime	≤0.03–>64	16	R	>64	R
Ceftriaxone	≤0.06–>128	128	R	>128	R
Aztreonam	≤0.06–>128	32	R	>128	R
Fosfomycin	≤0.25–>256	8	S	32	S
Imipenem	0.06–>64	0.25	S	>64	R
Meropenem	≤0.06–>128	≤0.06	S	128	R
Doripenem	≤0.03–>64	0.06	S	64	R
Biapenem	≤0.06–>128	0.25	No criteria	64	No criteria
Amikacin	0.12–>128	2	S	>128	R
Arbekacin	≤0.06–>128	1	No criteria	>128	No criteria
Gentamicin	≤0.06–>128	32	R	>128	R
Colistin	0.12–>32	0.5	No criteria	1	No criteria

*The interpretation of the MIC_50_ and MIC_90_ of all antibiotics tested for all pathogens was performed using the Clinical and Laboratory Standards Institute (CLSI) 2012 guidelines.

**Abbreviations:** S: susceptible, R: resistant, I: intermediate, amp/sulb: ampicillin/sulbactam, pip/taz: piperacillin/tazobactam, MIC: minimum inhibition concentration.

### P. aeruginosa

The MIC range, MIC_50_, and MIC_90_ of all antibiotics tested are presented in Table 4. The MIC_50_ was low, categorized as susceptible, for piperacillin/tazobactam, ceftazidime, aztreonam, doripenem, amikacin, gentamicin, and colistin (8/4, 8, 4, 2, 4, 2, and 1 µg/ml, respectively), while only colistin had low MIC_90_ which was in the susceptible range (2 µg/ml).

**Table 4:** Determination and interpretation of the MIC values of the antibiotics tested for *Pseudomonas aeruginosa* ** (n = 153).

Antibiotic	MIC range	MIC_50_ (µg/ml)	Interpretation*	MIC_90_ (µg/ml)	Interpretation*
Pip/tazo	0.5–>128	8/4	S	>128/4	R
Cefminox	64–>128	>128	No criteria	>128	No criteria
Cefmetazole	64–>128	>128	No criteria	>128	No criteria
Ceftazidime	0.25–>64	8	S	>64	R
Ceftriaxone	1–>128	64	No criteria	>128	No criteria
Aztreonam	0.12–>128	4	S	>128	R
Fosfomycin	1–>256	32	No criteria	256	No criteria
Imipenem	0.25- >64	8	R	>64	R
Meropenem	≤0.06–>128	4	I	>128	R
Doripenem	≤0.03–>64	2	S	>64	R
Biapenem	≤0.06–>128	2	No criteria	128	No criteria
Amikacin	0.25–>128	4	S	>128	R
Arbekacin	0.12–>128	1	No criteria	32	No criteria
Gentamicin	≤0.06–>128	2	S	>128	R
Colistin	0.12–16	1	S	2	S

*The interpretation of the MIC_50_ and MIC_90_ of all antibiotics tested for all pathogens was performed using the Clinical and Laboratory Standards Institute (CLSI) 2012 guidelines.

**Pseudomonas aeruginosa is intrinsically resistant to ampicillin/sulbactam and thus, MIC testing was not performed for this antibiotic.

**Abbreviations:** S: susceptible, R: resistant, I: intermediate, pip/taz: piperacillin/tazobactam, MIC: minimum inhibition concentration.

### *Acinetobacter* spp

The MIC range, MIC_50_, and MIC_90_ of all antibiotics tested are presented in Table 5. The MIC_50_ was low (0.5 µg/ml) and within the susceptible range only for colistin, while the MIC_90_ value was not low for any antibiotic.

**Table 5:** Determination and interpretation of the MIC values of the antibiotics tested for *Acinetobacter* spp.**(n = 354).

**Antibiotic**	**MIC range**	**MIC_50_ (µg/ml)**	**Interpretation** *	**MIC_90_ (µg/ml)**	**Interpretation** *
Amp/sulb	≤0.12–>256	32/16	R	128/64	R
Pip/tazo	≤0.06–>128	>128/4	R	>128/4	R
Cefminox	≤0.06–>128	64	No criteria	128	No criteria
Cefmetazole	0.25–>128	128	No criteria	>128	No criteria
Ceftazidime	≤0.03–>64	>64	R	>64	R
Ceftriaxone	≤0.06–>128	>128	R	>128	R
Aztreonam	≤0.06–>128	>128	No criteria	>128	No criteria
Imipenem	≤0.03–>64	32	R	>64	R
Meropenem	≤0.06–>128	32	R	>128	R
Doripenem	≤0.03–>64	32	No criteria	>64	No criteria
Biapenem	≤0.06–>128	32	No criteria	>128	No criteria
Amikacin	≤0.06–>128	>128	R	>128	R
Arbekacin	≤0.06–>128	>128	No criteria	>128	No criteria
Gentamicin	≤0.06–>128	>128	R	>128	R
Colistin	0.06–>32	0.5	S	64	R

*The interpretation of the MIC_50_ and MIC_90_ of all antibiotics tested for all pathogens was performed using the Clinical and Laboratory Standards Institute (CLSI) 2012 guidelines.

**Acinetobacter spp. is intrinsically resistant to fosfomycin and thus, MIC testing was not performed for this antibiotic.

**Abbreviations:** S: susceptible, R: resistant, I: intermediate, amp/sulb: ampicillin/sulbactam, pip/taz: piperacillin/tazobactam, MIC: minimum inhibition concentration.

In Table 6 the resistance profile of the included isolates to cefminox, biapenem, and arbekacin is presented in detail.

**Table 6:** Resistance profile of the included isolates to the study drugs.

Resistance to:	*Escherichia coli*	*Klebsiella* spp.	*Pseudomonas aeruginosa*	*Acinetobacter* spp.
***3^rd^ generation cephalosporins***				
Number of isolates	167	128	70	281
Cefminox MIC range	0.12–>128	0.25–>128	128–>128	0.25–>128
Cefminox MIC_50_	2	32	>128	64
Cefminox MIC_90_	>128	>128	>128	128
***Carbapenems***				
Number of isolates	48	74	76	292
Biapenem MIC range	0.06–>128	0.12–>128	0.5–>128	0.12–>128
Biapenem MIC_50_	16	8	16	32
Biapenem MIC_90_	64	128	>128	>128
***Aminoglycosides***				
Number of isolates	37	66	56	278
Arbekacin MIC range	2–>128	0.5–>128	0.5–>128	0.25–>128
Arbekacin MIC_50_	>128	>128	16	>128
Arbekacin MIC_90_	>128	>128	>128	>128

## Discussion

The main finding of the study is that fosfomycin was the only antibiotic that inhibited 90% of *E*. *coli* and *Klebsiella* spp. isolates, while colistin was the only antibiotic that inhibited 90% of *P*. *aeruginosa* isolates. In addition, 90% of *Acinetobacter* spp. isolates were not inhibited by any antibiotic tested.

Among the four antibiotics that were tested and are still not marketed in India, only fosfomycin seems to be a promising treatment option. However, the development of resistance during treatment with fosfomycin is an issue that has not been clarified yet and thus, fosfomycin should not be administered as monotherapy. [29] Interpretation of the antimicrobial susceptibility testing results could not be performed for arbekacin, cefminox, and biapenem. However, the MIC_90_ of these antibiotics was high for all four pathogens. Only biapenem was active against 50% of *E*. *coli* and *Klebsiella* spp. isolates (MIC_50_≤0.25 µg/ml for both pathogens) according to a previous study that attempted to suggest rational breakpoints for biapenem. [20] On the contrary, the MIC_50_ of arbekacin was very high for *Acinetobacter* spp. (>128 µg/ml) according to potential breakpoints suggested by one study (<2 µg/ml) [30].

It is noteworthy that piperacillin/tazobactam, cefmetazole, group 2 carbapenems, fosfomycin and amikacin were active against 50% of *E*. *coli* and *Klebsiella* spp. isolates but only fosfomycin retained this activity against 90% of these pathogens. Likewise, piperacillin/tazobactam, ceftazidime, aztreonam, doripenem, amikacin and gentamicin were active against 50% of *P*. *aeruginosa* isolates but colistin was the only antibiotic active against 90% of the isolates. The finding of the high *in vitro* activity of fosfomycin against *E*. *coli* has also been illustrated in one of our previous studies which evaluated the activity of fosfomycin against common uropathogens in India. [31] In general, the published literature suggests that fosfomycin might be an effective antibiotic against infections caused by MDR, including ESBL-producing, Enterobacteriaceae. [24], [32] Also, previous studies have shown high *in vitro* activity of colistin against MDR *P*. *aeruginosa* isolates, [33], [34] while treatment with colistin resulted in sufficient clinical effectiveness when administered to patients with severe infections due to MDR *P*. *aeruginosa* [35], [36], [37].

Regarding the antimicrobial resistance profile of the isolates included in this study, it is noteworthy that the MIC_50_ was high for extended-spectrum cephalosporins and aztreonam for *E*. *coli* and *Klebsiella* spp. isolates implying that these isolates may be possibly producers of ESBLs. Likewise, the MIC_50_ for imipenem and meropenem was high for *P*. *aeruginosa* and *Acinetobacter* spp. isolates and thus, these isolates may produce carbapenemases. It arises that high percentages of multidrug- or extensively drug-resistant Gram-negative pathogens are prevalent in this area of South India urging clinicians to consider alternative antibiotic options for the treatment of these infections.

Other interesting findings of this study are the high incidence of *Acinetobacter* spp. compared to other Gram-negative bacteria as well as the high antimicrobial resistance recorded among *Acinetobacter* spp. isolates. It is actually discouraging that only colistin was active against 50% of the isolates and 90% of the isolates were not inhibited by any antibiotic tested. Multidrug resistance of *Acinetobacter* spp. in India is a great concern addressed by previous studies, as well. [38], [39], [40] Tigecycline and colistin are treatment of choice against severe nosocomial infections due to MDR *Acinetobacter* spp. but emergence of resistance for both antibiotics has been reported in India leading to treatment deadlock. [41] In our study, antimicrobial susceptibility testing for tigecycline was not performed due to economic reasons.

The findings of the present study should be interpreted taking into consideration the limitation that the *in vitro* activity of tigecycline, which is one the most effective antibiotics used for the treatment of infections caused by *Acinetobacter* spp., was not tested. In addition, species identification of the *Klebsiella* and *Acinetobacter* isolates was not performed and therefore, the incidence and susceptibility of the individual species to the antibiotics tested could not be determined.

In conclusion, fosfomycin and colistin might be effective treatment options against infections caused by *E*. *coli* or *Klebsiella* spp. and *P*. *aeruginosa*, respectively, in India. However, clinical trials are needed to confirm the *in vitro* findings, especially before fosfomycin is introduced into clinical practice. The high antimicrobial resistance observed among *Acinetobacter* spp. isolates is a great concern which necessitates further investigation through studies evaluating the *in vitro* activity of tigecycline and antibiotic combinations. With regard to arbekacin, cefminox and biapenem, further microbiological studies are warranted to evaluate the activity of these antibiotics against clinical isolates in India.
